# Supplementary material for: Prebiotics Improve Blood Pressure Control by Modulating Gut Microbiome Composition and Function: A Systematic Review and Meta-Analysis
Source: Nutrients. 2025 Jul 30;17(15):2502. doi: 10.3390/nu17152502 (PMC12348426; doi:10.3390/nu17152502)
Supplement: Supplementary file 1 [file nutrients-17-02502-s001.zip › nutrients-3746587-supplementary.pdf]

Supplementary Materials

S1. Search Strategy

- **Question:**
  - How do dietary fibers affect blood pressure control by modulating select gut taxa and/or SCFA (acetate, butyrate, and propionate) production?
- **Inclusion/exclusion**
  - Include fiber/prebiotic as an intervention, and hypertension with either short-chain fatty acids or select bacterial taxa as a mechanism of action
  - Published in the last 10 years
  - Only interventional studies (manual screen)
- **Search terms**

PubMed

Date: 1/1/14 to 12/16/24

Results: 354

((Carbohydrates[Mesh] OR Carbohydrates[tiab] OR "Dietary Fiber"[Mesh] OR "Dietary Fiber"[tiab] OR "Dietary Fibers"[tiab] OR Prebiotics[Mesh] OR Prebiotics[tiab] OR Prebiotic[tiab] OR "Plant Fiber"[tiab] OR “Fiber mix"[tiab] OR Pectin[tiab] OR Pectins[Mesh] OR Inulin[Mesh] OR Inulin[tiab] OR Fructans[tiab] OR Fructans[Mesh] OR “Inulin-type fructans"[tiab] OR Oligofructose[tiab] OR “Oligofructose-enriched inulin"[tiab] OR Oligosaccharides[tiab] OR “Galacto-Oligosaccharides"[tiab] OR "Beta-Glucan"[tiab] OR "Beta-Glucans"[Mesh] OR "Resistant Starch"[Mesh] OR "Resistant Starch"[tiab] OR “RS1"[tiab] OR “RS2"[tiab] OR “RS3"[tiab] OR “RS4"[tiab] OR Rhamnose[Mesh] OR Rhamnose[tiab] OR “L-Rhamnose"[tiab] OR Arabinoxylan[tiab] OR arabinoxylan[Supplementary Concept] OR “Arabinoxylan Oligosaccharides"[tiab] OR "Guar Gum"[tiab] OR Orafti[tiab] OR "Synergy 1"[tiab] OR "Hi-Maize"[tiab] OR Novelose[tiab]))

AND

(((Firmicutes[Mesh] OR Firmicutes[tiab] OR Lactobacillaceae[Mesh] OR Lactobacillaceae[tiab] OR Ruminococcaceae[tiab] OR Lachnospiraceae[tiab] OR Bacteroidetes[Mesh] OR Bacteroidetes[tiab] OR Prevotella[tiab] OR Prevotella[Mesh] OR Bifidobacterium[Mesh] OR Bifidobacterium[tiab] OR "Bifidobacterium bifidum"[tiab] OR "Bifidobacterium bifidum"[Mesh] OR "Bifidobacterium longum"[tiab] OR “Bifidobacterium longum"[Mesh] OR "Bifidobacterium longum infantis"[tiab] OR "Bifidobacterium longum subspecies infantis"[Mesh] "Bifidobacterium breve"[tiab] OR "Bifidobacterium breve"[Mesh] OR Ruminococcus[tiab] OR Ruminococcus[Mesh] OR "Ruminococcus bromii"[tiab] OR "Ruminococcus bromii" [Supplementary Concept] OR "Ruminococcus gnavus"[tiab] OR “Ruminococcus gauvreauii"[tiab] OR Eubacterium[Mesh] OR Eubacterium[tiab] OR "Eubacterium rectale"[tiab] OR "Eubacterium hallii"[tiab] OR Blautia[tiab] OR "Blautia hydrogenotrophica"[tiab] OR Coprococcus[tiab] OR "Coprococcus eutactus"[tiab] OR "Coprococcus catus"[tiab] OR Roseburia OR Roseburia[tiab] OR "Roseburia intestinalis" [Supplementary Concept] OR "Roseburia intestinalis"[tiab] "Roseburia inulinivorans" [Supplementary Concept] OR "Roseburia inulinivorans"[tiab] OR "Roseburia hominis" [Supplementary Concept] OR "Roseburia faecis" [Supplementary Concept] OR "Roseburia cecicola" [Supplementary Concept] OR Faecalibacterium[Mesh] OR Faecalibacterium[tiab] OR "Faecalibacterium prausnitzii"[tiab] OR "Faecalibacterium duncaniae"[tiab] OR Lactobacillus[Mesh] OR Lactobacillus[tiab] OR Akkermansia[tiab] Akkermansia[Mesh] OR Veillonella[tiab])

OR

("Bacterial Taxa"[tiab] OR "Bacterial Composition"[tiab] OR "Microbial Composition"[tiab] OR "Gastrointestinal Microbiome"[Mesh]))

OR

(("short-chain fatty acids"[tiab] OR "short-chain fatty acids"[tiab] OR SCFAs[tiab] OR SCFA OR "Fatty Acids, Volatile"[Mesh] OR Acetate[tiab] OR Butyrate[tiab] OR Propionate[tiab])))

AND

((Hypertension[Mesh] OR Hypertension[tiab] OR "High Blood Pressure"[tiab] OR "Blood Pressure"[Mesh] OR “Hypertensive”[tiab] OR "Systolic Blood Pressure"[tiab] OR "Diastolic Blood Pressure"[tiab] OR "Arterial Pressure"[tiab] OR "Arterial Stiffness"[tiab]))

EMBASE

Date: 1/1/14 to 12/16/24

Results: 2882

(('carbohydrate'/exp OR carbohydrates:ti,ab OR 'dietary fiber'/exp OR 'dietary fiber':ti,ab OR 'dietary fibers':ti,ab OR 'prebiotic agent'/exp OR prebiotics:ti,ab OR prebiotic:ti,ab OR 'pectin'/exp OR pectin:ti,ab OR 'inulin'/exp OR inulin:ti,ab OR 'fructan'/exp OR fructans:ti,ab OR 'oligosaccharide'/exp OR oligosaccharides:ti,ab OR 'galacto oligosaccharides':ti,ab OR 'beta glucans'/exp OR 'resistant starch'/exp OR 'resistant starch':ti,ab OR rhamnose:ti,ab OR 'rhamnose'/exp OR arabinoxylan:ti,ab OR 'arabinoxylan'/exp OR 'guar gum'/exp OR 'guar gum':ti,ab))

AND

((('firmicutes'/exp OR firmicutes:ti,ab OR 'lactobacillaceae'/exp OR lactobacillaceae:ti,ab OR 'ruminococcaceae'/exp OR ruminococcaceae:ti,ab OR 'lachnospiraceae'/exp OR lachnospiraceae:ti,ab OR 'bacteroidetes'/exp OR bacteroidetes:ti,ab OR 'prevotella'/exp OR prevotella:ti,ab OR 'bifidobacterium'/exp OR bifidobacterium:ti,ab OR 'bifidobacterium bifidum'/exp OR 'bifidobacterium bifidum':ti,ab OR 'bifidobacterium longum'/exp OR 'bifidobacterium longum':ti,ab OR 'bifidobacterium breve'/exp OR 'bifidobacterium breve':ti,ab OR 'ruminococcus'/exp OR ruminococcus:ti,ab OR 'eubacterium'/exp OR eubacterium:ti,ab OR 'blautia'/exp OR blautia:ti,ab OR 'coprococcus'/exp OR coprococcus:ti,ab OR 'roseburia'/exp OR roseburia:ti,ab OR 'faecalibacterium'/exp OR faecalibacterium:ti,ab OR 'faecalibacterium prausnitzii'/exp OR 'faecalibacterium prausnitzii':ti,ab OR 'lactobacillus'/exp OR lactobacillus:ti,ab OR 'akkermansia'/exp OR akkermansia:ti,ab OR 'veillonella'/exp OR veillonella:ti,ab)

OR

('bacterial taxa':ti,ab OR 'bacterial composition':ti,ab OR 'microbial composition':ti,ab OR 'gastrointestinal microbiota'/exp))

OR

(('short chain fatty acids':ti,ab OR scfas:ti,ab OR scfa:ti,ab OR 'volatile fatty acid'/exp OR acetate:ti,ab OR butyrate:ti,ab OR propionate:ti,ab)))

AND

(('hypertension'/exp OR hypertension:ti,ab OR 'high blood pressure':ti,ab OR 'blood pressure'/exp OR hypertensive:ti,ab OR 'systolic blood pressure':ti,ab OR 'diastolic blood pressure':ti,ab OR 'arterial pressure':ti,ab OR 'arterial stiffness':ti,ab))

Risk of Bias Assessment:

Table S1. Animal Study Risk of Bias

Study	Sequence Generation	Baseline Characteristics	Allocation Concealment	Random Housing	Blinding (Intervention)	Random Outcome Assessment	Blinding (Outcome)	Incomplete outcome Data	Selective Outcome Reporting	Other Sources of Bias
Avellaneda-Franco et al. (2024)	?	+	?	?	?	?	?	N.A.	+	+
Bulut et al. (2024)	?	+	?	?	?	?	?	N.A.	+	+
Ganesh et al. (2018)	?	+	N.A.	?	?	?	-	N.A.	+	+
Han et al. (2021)	?	+	?	?	?	?	?	N.A.	+	+
Hsu et al. (2018)	?	+	?	?	?	?	?	N.A.	+	+
Kay et al. (2020)	?	+	?	?	?	?	?	N.A.	+	+
Le it al (2022)	?	+	?	?	?	?	?	N.A.	+	+
Marque et al. (2017)	?	+	?	?	?	?	?	N.A.	+	+
O'Connor et al. (2020)	?	+	?	?	?	?	?	N.A.	+	+
Xie et al. (2022)	?	?	?	?	?	?	?	N.A.	?	+
Zhang et al. (2019)	?	+	?	?	?	?	?	N.A.	+	+
Chao et al. (2022)	?	+	?	?	?	?	?	N.A.	+	+

+ indicates low risk of bias; (-) indicates high risk of bias; (N.A.) not applicable; (?) indicates unclear risk of bias

Table S2. Human Study Risk of Bias

Study	D1	D2	D3	D4	D5	D6	D7	Overall
Cao et al. (2022)	Some concerns	Some concerns	Low	Low	Low	None	None	Low
Sos Santos Fechine et al. (2021)	Some concerns	High	Low	Some concerns	Low	None	None	High
Hiel et al. (2020)	Low	Low	Low	Low	Low	None	None	Low
Jama et al. (2022)	Low	Low	Low	Low	Low	None	None	Low
Roshanravan et al. (2023)	Low	Some concerns	Low	Low	Low	None	None	Low
Zue et al. (2021)	Low	Some concerns	Low	Low	Low	None	None	Low
Vijay et al. (2021)	Low	Low	Low	Low	Low	Low	Low	Low
